# Comparison of efficacy of Retzius-sparing radical prostatectomy versus standard radical prostatectomy in the treatment of prostate cancer: a systematic review and meta-analysis

**DOI:** 10.3389/fonc.2025.1547687

**Published:** 2025-05-14

**Authors:** Weilun Gong, Junfeng Yan, Yilun Cui, Duojie Zhang, Yinfeng Ma

**Affiliations:** ^1^ Department of Urology, The Second Affiliated Hospital of Zhejiang Chinese Medicine University, Hangzhou, Zhejiang, China; ^2^ Department of Urology, Zhejiang Hospital, Hangzhou, Zhejiang, China; ^3^ Department of Urology, The Third Affiliated Hospital of Wenzhou Medical University, Wenzhou, Zhejiang, China

**Keywords:** radical prostatectomy, Retzius-sparing, robot-assisted, prostate cancer, urinary continence

## Abstract

**Objective:**

To compare the efficacy of Retzius-sparing robot-assisted radical prostatectomy (RS-RARP) versus standard robot-assisted radical prostatectomy (S-RARP) in treating prostate cancer patients regarding urinary continence (UC) recovery, oncological control, and other complications.

**Methods:**

An electronic search was performed on four databases with no restrictions on the language up to May 16^th^, 2024. The main outcomes were UC recovery positive, positive surgical margin (PSM), biochemical recurrence (BCR) and postoperative complications. Result robustness was enhanced based on the RoB and quality assessments.

**Results:**

The final analysis included 3 randomized controlled trials, 2 prospective studies, and 4 retrospective studies. According to quantitative results, RS-RARP improved the UC recovery rates at catheter removal (OR=11.33, 95% CI=[1.29-99.69], P=0.03), at 1 month (OR=14.18, 95% CI=[1.34-150.44], P=0.03), 3 months (OR=3.64, 95% CI=[1.94-6.83], P<0.00001), 6 months (OR=3.18, 95% CI=[1.62-6.22], P=0.0007), but failed to present a better continence recovery rate at 12 months (OR=2.30, 95% CI=[0.77-6.85], P=0.14) postoperatively. The RS-RARP group presented higher overall PSM rates (OR=1.51, 95% CI=[1.15-1.98]) and PSM rates in ≥ pT3 tumors (OR=1.81, 95% CI=[1.18-2.77], P=0.006) versus the S-RARP group. Furthermore, the two groups did not present obviously different BCR rates (OR=0.58, 95% CI=[0.20-1.67], P=0.31), operating time (WMD=10.41 min, 95% CI=[-2.82-23.65], P=0.12), intraoperative estimated blood loss (WMD=-15.97 mL, 95% CI=[-41.53-9.58], P=0.22), serious postoperative complications (OR=1.04, 95% CI=[0.50-2.13], P=0.10).

**Conclusions:**

Our meta-analysis revealed that although RS-RARP demonstrated accelerated urinary continence recovery, it showed a tendency toward higher PSM rates in patients with ≥pT3 tumors.

## 1 Introduction

An innovative surgical technique, named the Bocciardi technique, was first proposed by Galfano et al. in 2010, for robot-assisted radical prostatectomy (RARP) in which the prostate is approached posteriorly, together with an intact retzius space (1). Compared with the standard robot-assisted radical prostatectomy (S-RARP), an anterior surgical approach involving sequential entry into the Retzius space, incision of the endopelvic fascia, controlled division of the puboprostatic ligament, transection of the dorsal venous complex (DVC), and complete exposure of the prostate, the Retzius-sparing robot-assisted radical prostatectomy (RS-RARP) approach can preserve the endopelvic fascia, arcus tendinous, puboprostatic ligament, the detrusor apron, Santorini plexus, and deep dorsal vein plexus (2). Therefore, it facilitates earlier UC recovery and preserves erectile function, as evidenced in various randomized controlled trials (RCTs) and prospective studies (3–11). However, other studies found no benefit of the RS-RARP on longer-term UC recovery and potency recovery (12). Furthermore, its feature of preserving more anterior structures in the retzius space means a smaller workspace and fewer landmarks for surgeons. These factors may not only extend the surgeons’learning curve, but also results in larger positive surgical margins (PSM) (13, 14). PSM independently predicts the biochemical recurrence and primarily determines if salvage treatment is needed (15). Proper and early detection of biochemical recurrence rate (BCR) can timely guide the application of curative-intent salvage therapies. Therefore, the study aimed at comparing the performance of the two approaches in treating prostate cancer patients through a long-term follow-up from the perspectives of UC recovery and oncological control.

## 2 Materials and methods

The meta-analysis adhered to the Preferred Reporting Items for Systematic Reviews and Meta-Analyses guidelines. Two investigators identified relevant studies, screened and extracted the data as well as conducted validity assessment independently, and upon the emergence of any discrepancies in the data, a third reviewer would be invited to participate in the consultation.

### 2.1 Search strategy and selection criteria

The Pubmed, EMBASE, WoS and Cochrane Library were subjected to a systematic electronic literature search up to May 16th, 2024. The primary search strategy used the terms of: (((prostate) OR (prostatic)) AND ((((neoplasm) OR (cancer)) OR (carcinoma)) OR (malignancy))) AND ((((Retzius-sparing) OR (RARP)) OR (Retzius preservation)) OR (Bocciardi approach)) AND ((humans[Filter]) AND (all adult[Filter])).

To minimize the bias arising from the small number and sample size of RCTs, we included non-RCT studies in the meta-analysis. Consequently, the inclusion criteria were relaxed: (1) studies that enrolled adults with prostate cancers and (2) those that compared RS-RARP with S-RARP. Those found to be reviews, meta-analyses, replies, comments, case reports, conference reports, conference abstract, notes, book chapters, and non-comparative studies were excluded.

### 2.2 Risk-of-bias and study quality assessment

The risk of bias (RoB) among the identified RCTs and observational studies was determined by virtue of RoB 2.0 and ROBINS-I (16, 17). The RoB 2.0 comprises 5 domains, scored as “low”, “some concerns”, or “high”. In ROBINS-I, the RoB is scored as “low”, “moderate”, “serious”, or “critical”. All studies were rated by the Grades of Recommendation, Assessment, Development and Evaluation (GRADE), classified as “very low”, “low”, “moderate”, and “high” (18).

### 2.3 Data extraction and statistical analysis

The baseline characteristics extracted from each study included age, BMI, prostate size, pre-operative prostate-specific antigen, clinical stage, and pre-operative Gleason score. The oncological outcomes comprised the pathological stage (pT) and PSM, whereas functional outcomes included continence and potency recovery. We also recorded the perioperative operative time, EBL, and postoperative complication rates.

Statistical analysis relied on the Review Manager v5.3 software (Cochrane Collaboration, Oxford, UK). Presentation of continuous variables followed the mean ± standard deviation (SD) format, and that of categorical variables followed frequency and percentage format. If a continuous variable presentation followed the median and interquartile range (IQR) format, the numerical scales were employed to estimate data from such variables (19–22). The Weighted Mean Difference (WMD) with 95% CIs served as a summary measure for continuous data, and odds ratios (ORs) with 95% CIs were used for dichotomous data. A random-effect (RE) model explained the heterogeneity. Study heterogeneity was tested by calculating the P value and the I2 statistic, with a P value < 0.10 indicating high heterogeneity (23). P < 0.05 reported statistical significance.

## 3 Results

**Figure 1:**
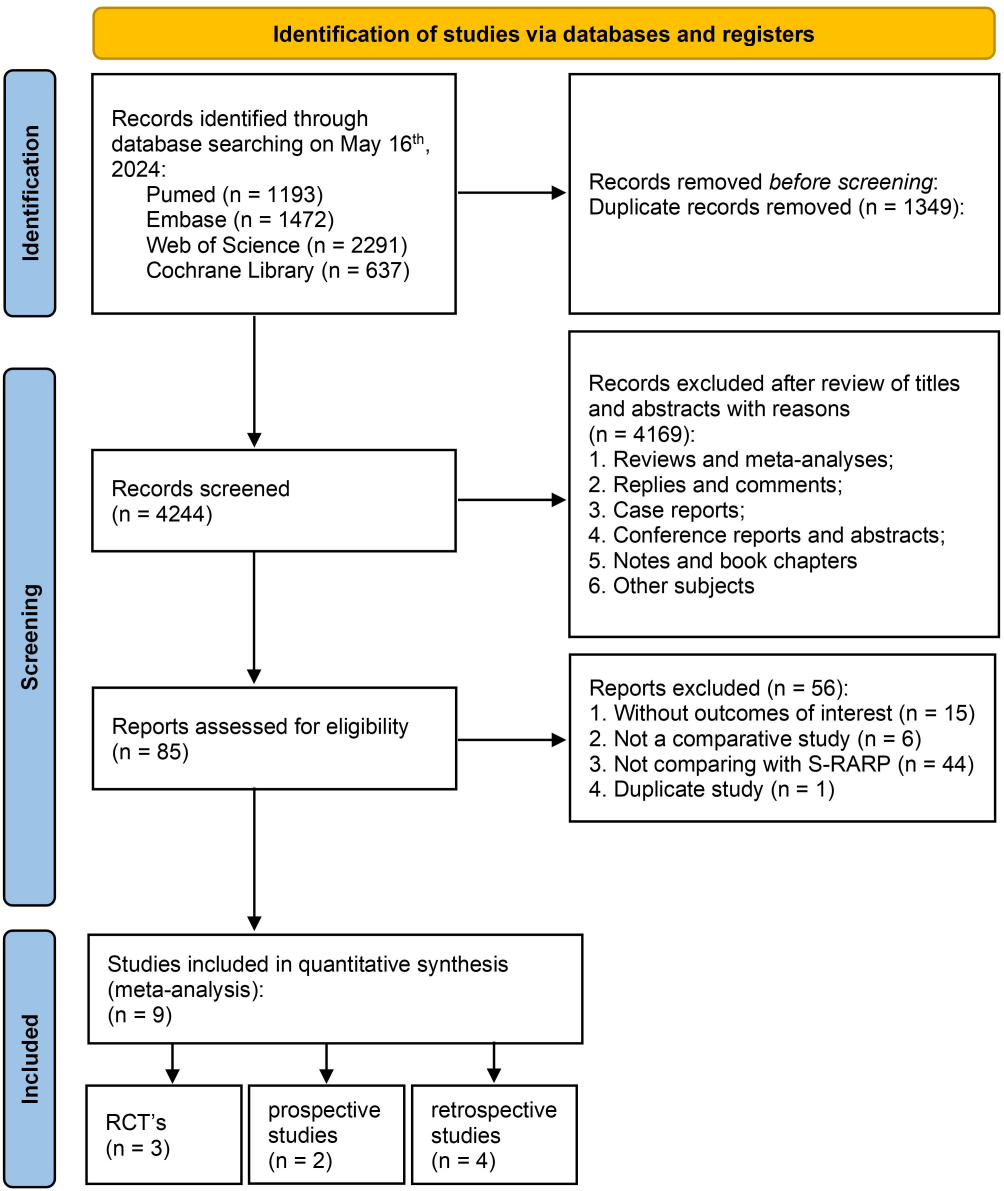
PRISMA flowchart.

Nine relevant studies were identified from four electronic databases, including 3 RCTs, 2 prospective studies, and 4 retrospective studies. **Figure 1** illustrates the PRISMA (Preferred Reporting Items for Systematic Reviews and Meta-analyses) flowchart regarding the search strategy. For duplicate studies reporting the same patient populations, the latest study was included.

### 3.1 Characteristics of included studies

**Figure 2:**
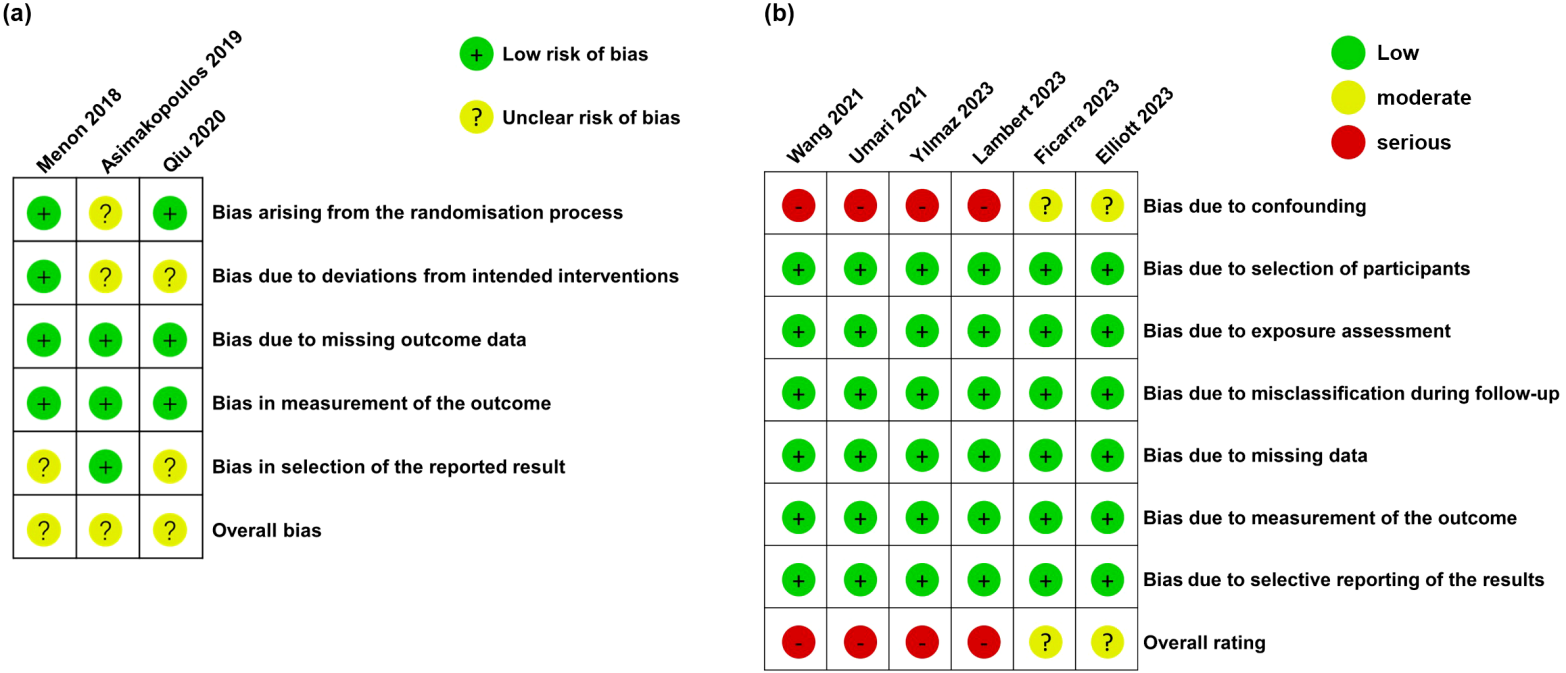
Risk of bias among the enrolled RCTs **(a)** and non-randomized studies **(b)**.

Based on the results of the RoB 2.0 test, 3 RCTs were considered to exhibit a high RoB (**Figure 2a**). The RoB results for 6 observational studies were assessed by ROBINS-I, and results are presented in **Figure 2b**. The 4 observational studies were considered to exhibit a high RoB, whereas another 2 observational studies showed a moderate RoB. **Table 1** lists the level of evidence in all studies (6–9, 11, 12, 24–26). Overall, included studies involved 775 patients receiving RS-RARP and 775 patients receiving S-RARP.

**Table 1:** Characteristics of included studies.

Study	Level of evidence	No. of patients		Age (year)		BMI (kg/m^2^)		PSA (ng/ml)		Gleason score (RS-RARP)			Gleason score (S-RARP)		
		RS	S	RS	S	RS	S	RS	S	≥6	7	≥8	≥6	7	≥8
Menon 2018 (6)	⊕⊕⊕⊙Moderate*	60	60	61.0 ± 9.1	61.5 ± 8.4	28.2 ± 3.4	28.4 ± 3.4	5.9 ± 2.1	5.5 ± 2.7	18 (30.0)	42 (70.0)	0 (0)	20 (33.0)	30 (67.0)	0 (0)
Asimakopoulos 2019 (7)	⊕⊕⊕⊙Moderate*	39	40	66.0 ± 5.3	65 ± 5.8	NR	NR	6.9 ± 2.2	7.0 ± 0.7	27 (69.2)	12 (30.7)	0 (0)	28 (70.0)	8 (20.0)	4 (10.0)
Qiu 2020 (8)	⊕⊕⊕⊕High	55	55	66.9 ± 6.9	67.0 ± 7.6	24.3 ± 3.1	23.9 ± 2.5	10.0 ± 7.2	9.0 ± 1.5	24 (43.6)	21 (38.1)	10 (18.2)	20 (36.4)	23 (41.8)	12 (21.8)
Wang 2021 (24)	⊕⊙⊙⊙Very low^‡^	40	52	68.4 ± 5.7	67.8 ± 5.8	23.8 ± 3.1	24.0 ± 2.8	11.9 ± 4.9	12.2 ± 4.7	NR	NR	NR	NR	NR	NR
Umari 2021 (12)	⊕⊕⊕⊙Moderate*	282	201	62.8 ± 7.5	60.4 ± 7.5	26.7 ± 4.0	26.7 ± 3.5	8.8 ± 16.7	10.4 ± 16.6	44 (15.0)	201 (78.0)	37 (13.0)	4 (2.0)	167 (83.1)	30 (14.9)
Yılmaz 2023 (25)	⊕⊙⊙⊙Very low^‡^	60	60	65.9 ± 5.4	66.2 ± 5.3	27.1 ± 3.9	26.8 ± 3.9	22.2 ± 40.9	22.6 ± 41.5	30 (50.0)	24 (40.0)	6 (10.0)	32 (53.3)	23 (38.4)	5 (8.4)
Lambert 2023 (9)	⊕⊙⊙⊙Very low^‡^	100	100	65.6 ± 6.8	67.4 ± 8.3	26.5 ± 3.5	26.3 ± 3.3	7.8 ± 3.9	8.5 ± 3.5	18 (18.0)	54 (54.0)	28 (28.0)	15 (15.0)	66 (66.0)	19 (19.0)
Ficarra 2023 (11)	⊕⊕⊕⊙Moderate*	102	105	65.1 ± 7.5	66.3 ± 7.5	26.0 ± 3.0	26.8 ± 3.6	7.1 ± 2.8	8.0 ± 3.8	49 (48.0)	44 (43.1)	9 (8.8)	43 (41.0)	46 (43.8)	16 (15.3)
Elliott 2023 (26)	⊕⊙⊙⊙Very low^§^	37	37	65.7 ± 4.9	65.4 ± 5.0	27.9 ± 3.7	28.9 ± 5.3	8.9 ± 4.9	7.2 ± 3.3	6 (16.2)	21 (56.7)	10 (27.0)	7 (18.9)	22 (59.4)	8 (21.6)

Continuous variable presentation followed mean ± SD format, and categorical variable presentation followed frequency and percentage format. PSA, prostate-specific antigen; S-RARP, standard robot-assisted radical prostatectomy; RS-RARP, Retzius-sparing robot-assisted radical prostatectomy; RCTs, randomized controlled trials. Moderate level of evidence: we are moderately confident in the effect estimate: the true effect is likely to be close to the estimate of the effect, but there is a possibility that it is substantially different. Low level of evidence: our confidence in the effect estimate is limited: the true effect may be substantially different from the estimate of the effect. Very low level of evidence: we have very little confidence in the effect estimate: the true effect is likely to be substantially different from the estimate of effect. ^*^Upgraded one level due to objective findings, rigorous methodology, large effect size. ^‡^Downgraded one level due to confounding bias for studies without describing potential confounders control. ^§^Downgraded two levels due to the small number of events and the wide CI.

### 3.2 Continence recovery

**Figure 3:**
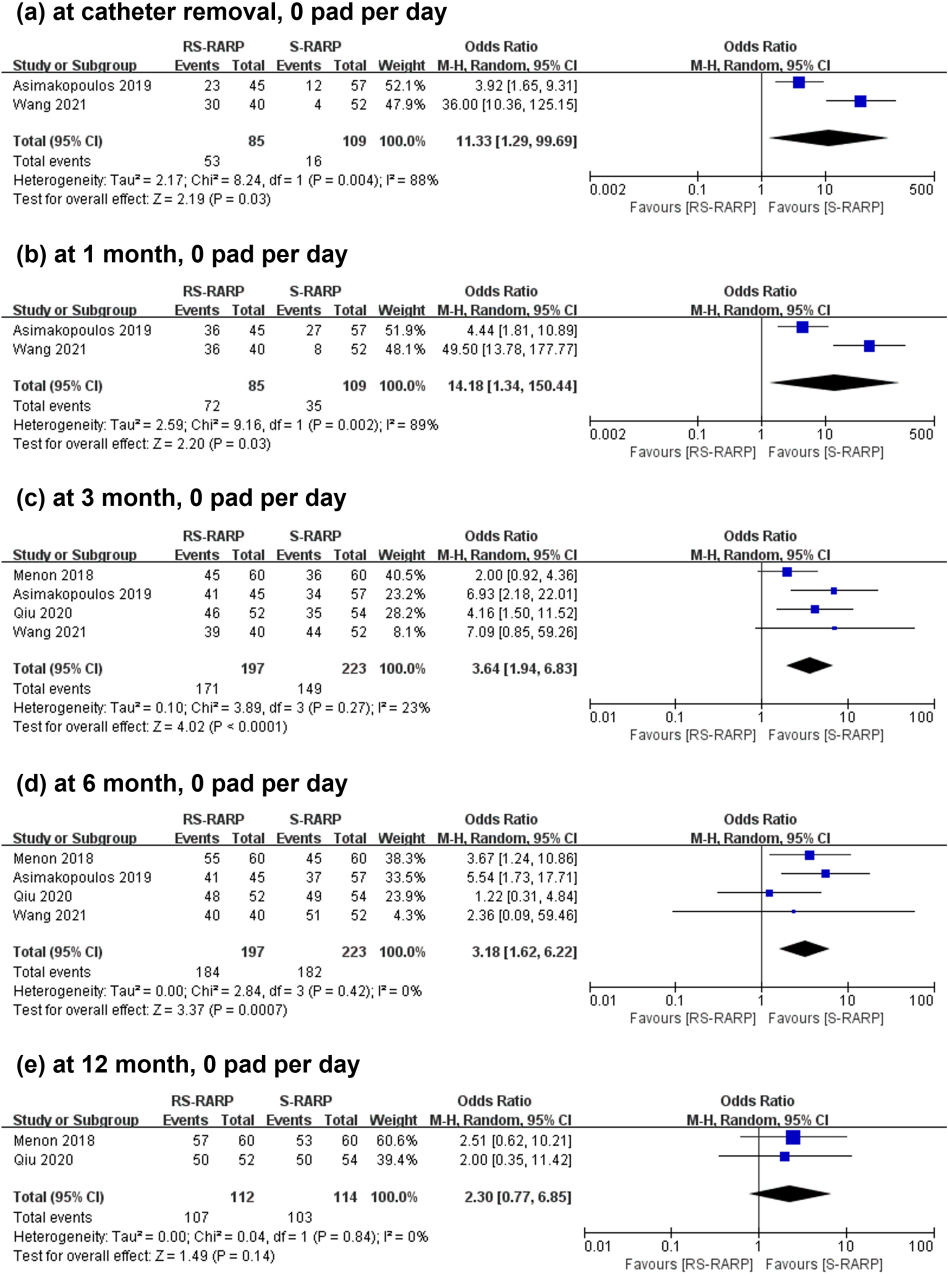
Forest plot showing the continence recovery rates post-operatively at catheter removal **(a)**, 1 month **(b)**, 3 months **(c)**, 6 months **(d)**, 12 months **(e)** between the RS-RARP group and the S-RARP group. M-H, Mantel-Haenszel.

According to the strict continence definition (use of 0 pad each day), we found that the RS-RARP group showed a better cumulative continence recovery rate (CRR) at catheter removal (OR=11.33, 95% CI=[1.29-99.69], P=0.03, **Figure 3a**), 1 month (OR=14.18, 95% CI=[1.34-150.44], P=0.03, **Figure 3b**), 3 months (OR=3.64, 95% CI=[1.94-6.83], P<0.00001, **Figure 3c**), 6 months (OR=3.18, 95% CI=[1.62-6.22], P=0.0007, **Figure 3d**), but did not show a better CRR at 12 months (OR=2.30, 95% CI=[0.77-6.85], P=0.14, **Figure 3e**) postoperatively. However, when continence was defined by 0–1 pad per day, significant differences in 12-month continence recovery rates were found between the groups (OR=12.08, 95% CI=[1.59-91.72], P=0.02, **Supplementary Figure S1**).

### 3.3 PSMs and BCR

**Figure 4:**
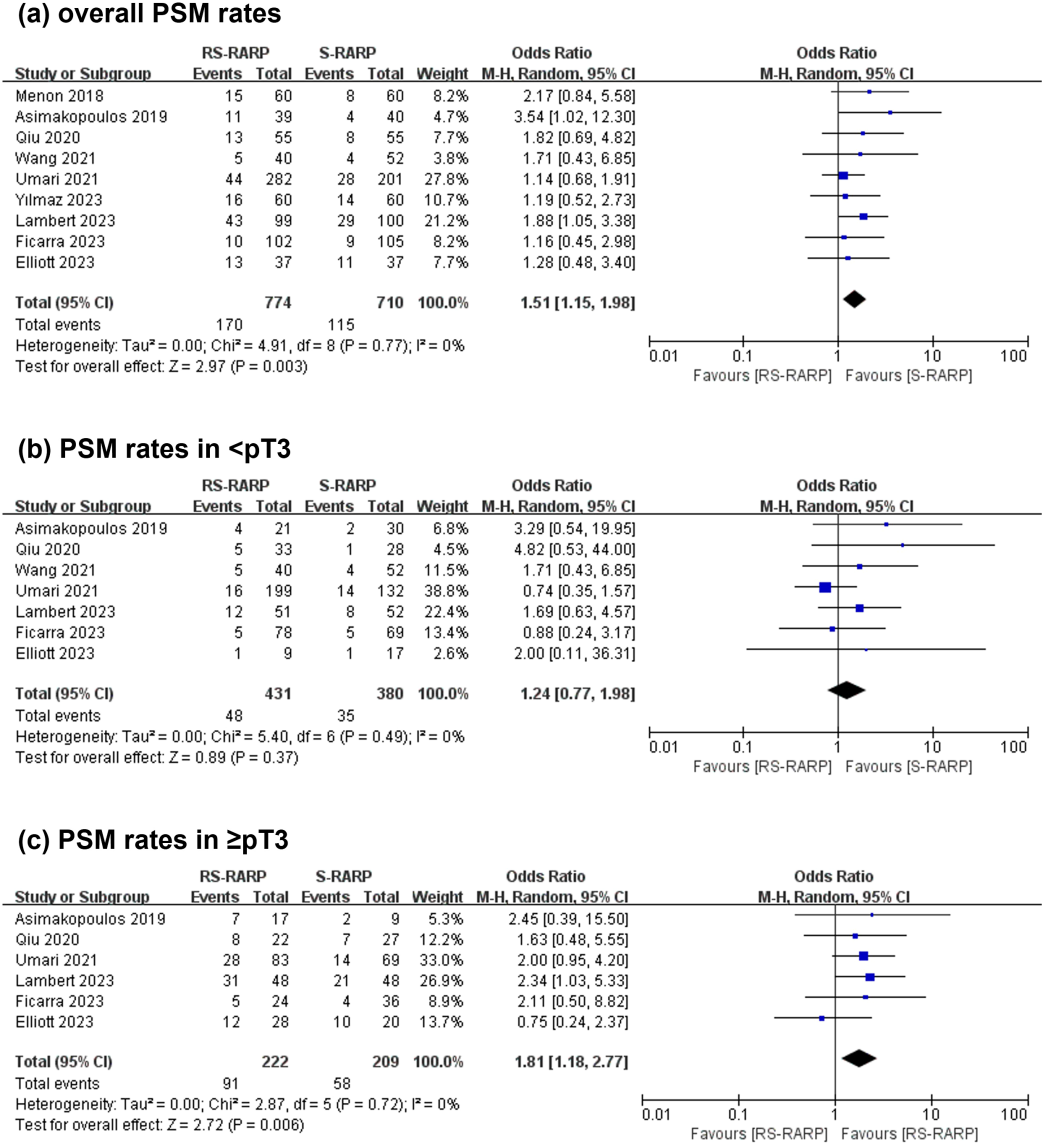
Forest plot showing the PSM rates post-surgery between the RS-RARP and the S-RARP groups. **(a)** Meta-analysis for the overall PSM rates. **(b)** Meta-analysis for the < pT3 PSM rates. **(c)** Meta-analysis for ≥ pT3 PSM rates.

**Figure 5:**
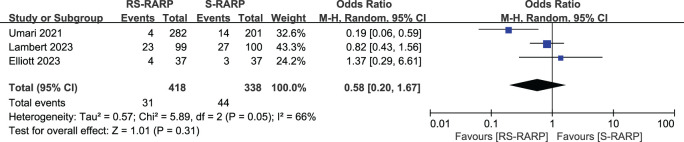
Forest plot illustrating the BCR rates post-operatively between the two groups.

The RS-RARP group exhibited higher overall PSM rates (170 of 774 cases, 21.96%) versus the S-RARP group (115 of 710 cases, 16.20%) (OR=1.51, 95% CI=[1.15-1.98], P=0.003, **Figure 4a**). For < pT3 tumors, the two groups did not present obviously different PSM rates (OR=1.24, 95% CI=[0.77-1.98], P=0.37, **Figure 4b**). However, for≥ pT3 tumors, the RS-RARP group showed remarkably higher PSM rates versus the S-RARP group (OR=1.81, 95% CI=[1.18-2.77], P=0.006, **Figure 4c**). Furthermore, three studies reported the BCR rates at 1-year follow-up, and no significant difference in BCR rates was observed between the two groups (OR=0.58, 95% CI=[0.20-1.67], P=0.31, **Figure 5**).

### 3.4 Operating time, EBL and complications

**Figure 6:**
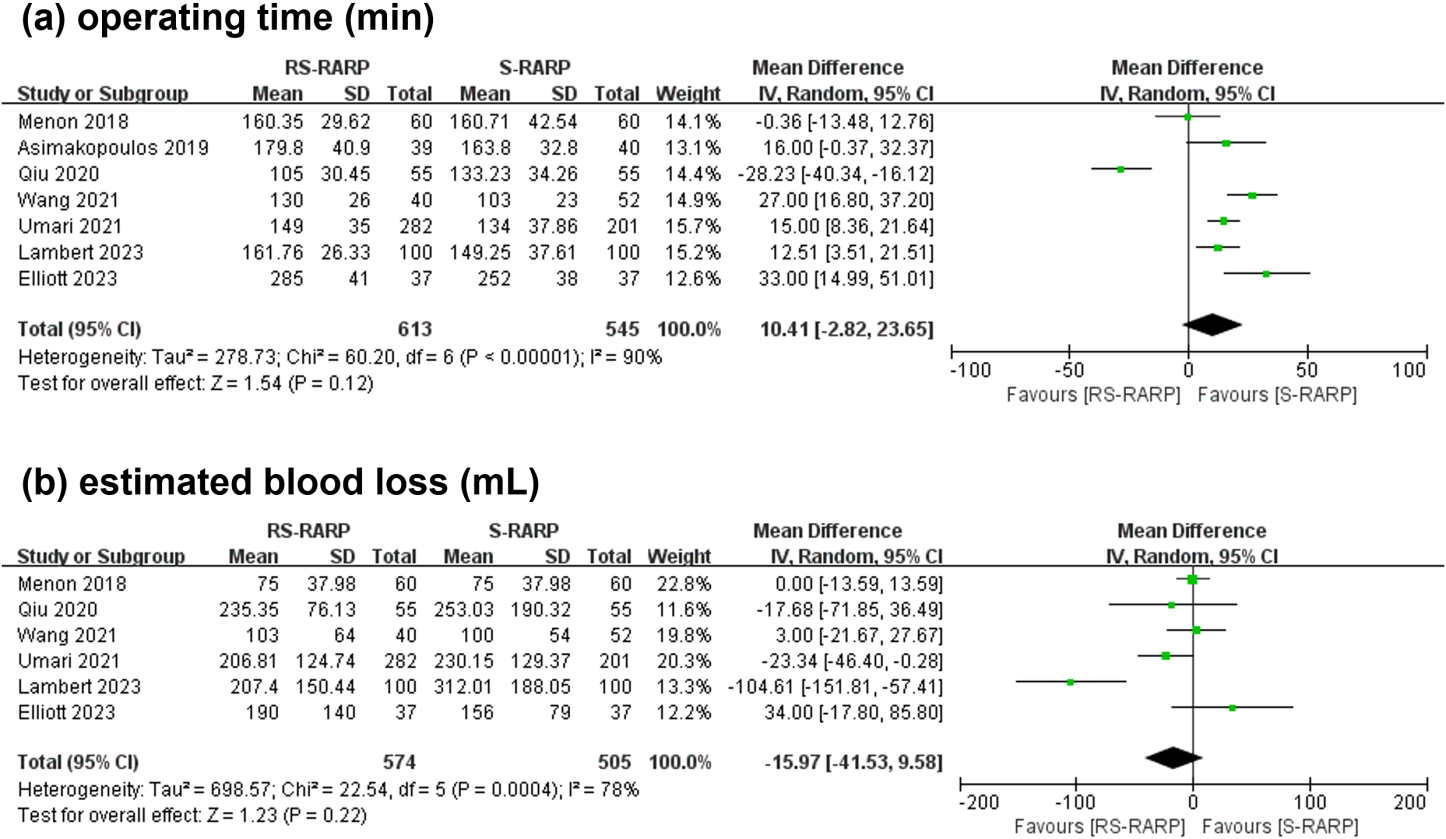
Forest plot showing the intraoperative operating time **(a)** and estimated blood loss **(b)** between the two groups.

**Figure 7:**
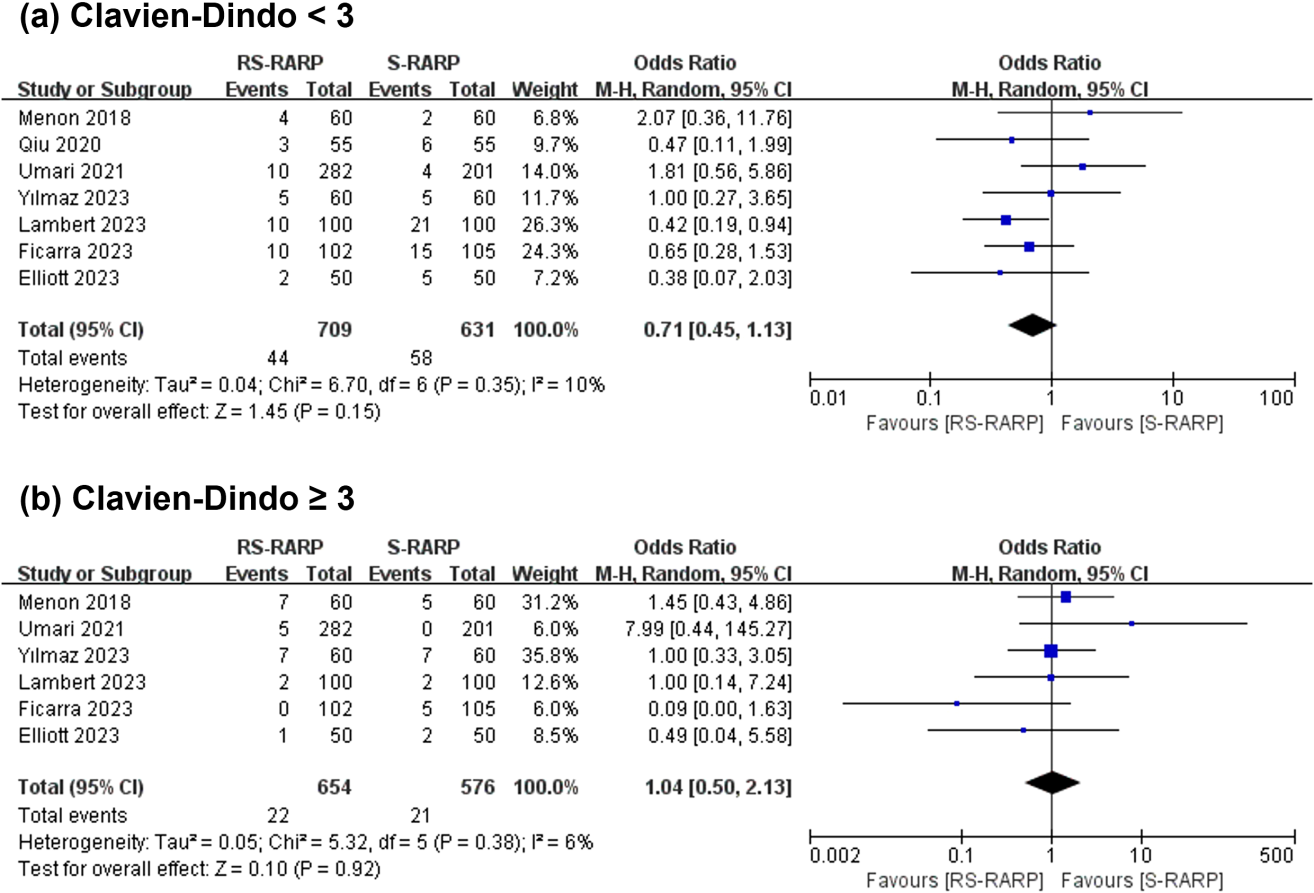
Forest plot showing the postoperative complications between the two groups.

Seven studies reported operating time, and 6 studies provided data for the console time separately. The analysis revealed that the two groups did not present obviously different operating time (WMD=10.41 min, 95% CI=[-2.82-23.65], P=0.12, **Figure 6a**) and EBL intraoperatively (WMD=-15.97 mL, 95% CI=[-41.53-9.58], P=0.22, **Figure 6b**). In addition, 7 studies reported post-operative complications, graded by the standardized Clavien-Dindo classification. Notably, grade < 3 (OR=0.71, 95% CI=[0.45-1.13], P=0.15, **Figure 7a**) or grade ≥ 3 (OR=1.04, 95% CI=[0.50-2.13], P=0.10, **Figure 7b**) were considerably no different between the two groups.

## 4 Discussion

As an innovative surgical technique, RS-RARP can protect the Retzius space, including the neurovascular bundle, which promotes early recovery of continence and erectile function. However, the long-term oncological efficacy and 12-month continence recovery for patients receiving this procedure are poorly understood (14, 27, 28). Therefore, the available evidence on the impact of RS-RARP on long-term continence recovery and oncological control was summarized by the systematic review. According to the result, the RS-RARP group exhibited a higher CRR versus the S-RARP group at catheter removal, 1 month, 3 months and 6 months postoperative with continence defined as 0 pad per day. However, the RS-RARP group also demonstrated a higher PSM rate versus the S-RARP group. Moreover, the two groups did not present significant different BCR rates, operation time, EBL, and complications.

Continence recovery is an important endpoint following radical prostatectomy, and can affect patients’ life quality. Most systematic reviews and meta-analyses found that due to its property of maintaining the normal pelvic anatomy, the RS-RARP had better early continence recovery versus the S-RARP technique. In our analysis, the RS-RARP group had higher continence rates after catheter removal and at 1, 3, and 6 months relative to the S-RARP group. However, whether continence recovery at 12 months is superior following the RS-RARP procedure remains to be determined. Several studies have demonstrated basically similar CRRs after surgery at 12 months between the two groups, while others found significant differences at 12 months between the two groups. In our analysis, when continence was defined as 0–1 safety pad per day, the RS-RARP group exhibited a higher CRR versus the S-RARP group (14, 27–30). However, the two groups showed a comparable CRR at 12 months with continence defined as 0 pad per day. We suggest that RS-RARP may improve the 1-year continence recovery, but this benefit is marginal.

Notably, surgical innovations for the management of solid cancer are evaluated based on their ability to provide sufficient oncological control. The PSM rate is considered a predictor for BCR, which indicates a poor oncological outcome for localized prostate cancer post-surgery. Conflicting evidence exists on PSM predictors. Several studies found PSM rates possibly because of the surgeons’ learning curve for the RS-RARP technique (15, 27). Several studies have identified a potential association between PSM occurrence in RS-RARP and the evolving competence of surgeons during the initial implementation phase of this robotic approach (31, 32). Nevertheless, its long-term therapeutic effects show variability depending on additional clinical parameters (15). In our analysis, the RS-RARP group exhibited dramatically higher overall PSM rates and PSM rates in localized ≥ pT3 tumors versus the S-RARP group. But the RS-RARP group did not exhibit a significant increase PSM rates in < pT3. It is likely that the difference between the two approaches may reflect by the higher proportion of pT3 disease in RS-RARP, as a higher tumor extension will result in increased risk of PSM (13, 33). Another factor to considered is the anatomic structure of the prostatic capsule. Xu J-N et al. found that anterior tumors are associated with higher PSM rates (34). However, in this analysis, the two groups showed comparable BCR rates, similar to findings from previous meta-analyses (14, 27, 28).

Moreover, the RS-RAPP group and the S-RAPR group did not present obviously different intraoperative conditions or postoperative complications. Regarding console time, one study by Lim et al. reported that the RS-RARP group had shorter console time versus the matched S-RARP group (3). Seven studies reported no differences in console time between the two groups, which may be due to the learning curve factor (35). Regarding postoperative complications, Lambert et al. found that the RS-RARP group had fewer complications relative to the S-RARP group, but no difference in Clavin-Dindo grade ≥ 3 between the two groups (9).

Despite abovementioned important findings, this study had several limitations. Firstly, although all evidence in the existing literature were included in our analysis, the number of RCTs and prospective studies was small. In addition, the sample size of included studies was relatively small. Secondly, even though we standardized the definitions and measurements of outcomes, methodological heterogeneity cannot be ruled out. Differences in surgeons’ learning curve of RS-RARP may also introduce bias. Thirdly, because different methods were used to assess erectile dysfunction without comparative date, the present study did not include sexual function recovery data.

Additionally, for the anterior approach several techniques (e.g. hood and sleeve technique) have been published describing the preservation of the anterior and anterolateral periprostatic structures (e.g. dorsovascular complex and pubovesical ligaments). RCTs comparing these techniques with RS-RARP are lacking, but a positive influence on the continence can be assumed.

In conclusion, RS-RARP achieves immediate continence recovery, but may be associated with an elevated risk of PSM rates in ≥ pT3 tumors. Reassuringly, the two approaches demonstrated comparable 1-year BCR rates. In future, the long-term oncological outcomes as well as the erectile function recovery of the two techniques should be confirmed in high-quality, multicenter RCTs.

## Data Availability

The original contributions presented in the study are included in the article/**Supplementary Material**. Further inquiries can be directed to the corresponding author.

## Supplementary material

Supplementary Figure 1 Forest plot illustrating continence recovery rates post-surgery at 12 months between the two groups when defined as 0–1 safety pad per day.
