# Supplementary material for: Comparison of efficacy of Retzius-sparing radical prostatectomy versus standard radical prostatectomy in the treatment of prostate cancer: a systematic review and meta-analysis
Source: Front Oncol. 2025 May 14;15:1547687. doi: 10.3389/fonc.2025.1547687 (PMC12120473; doi:10.3389/fonc.2025.1547687)
Supplement: Supplementary Figure 1 — Forest plot illustrating continence recovery rates post-surgery at 12 months between the two groups when defined as 0–1 safety pad per day. [file DataSheet1.docx]

Supplementary Material

# Supplementary Figure S1


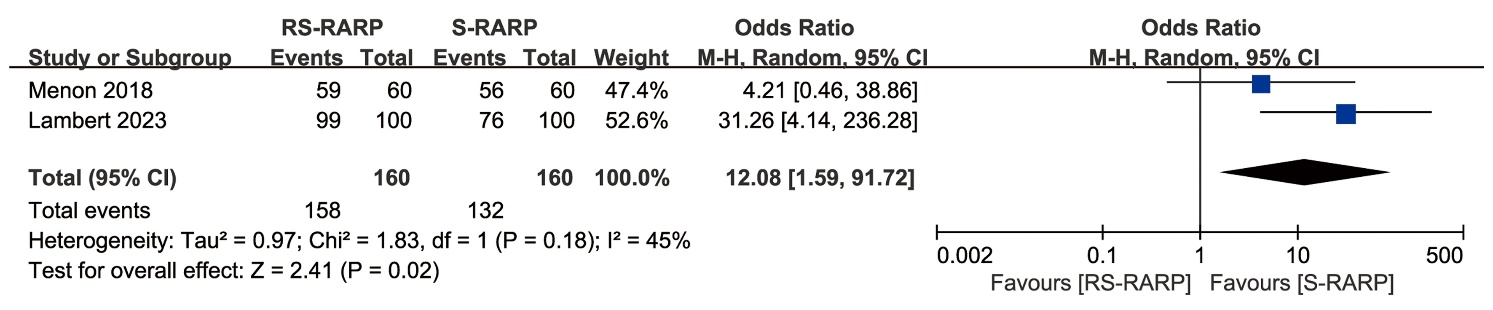


**Supplementary Figure 1.** Forest plot illustrating continence recovery rates post-surgery at 12 months between the two groups with continence defined as 0-1 safety pad per day. M-H, Mantel-Haenszel.
